# Reliability assessment of hyperspectral imaging with the HyperView™ system for lower extremity superficial tissue oxygenation in young healthy volunteers

**DOI:** 10.1007/s10877-021-00698-w

**Published:** 2021-04-12

**Authors:** Simone F. Kleiss, Kirsten F. Ma, Mostafa El Moumni, Richte C. L. Schuurmann, Clark J. Zeebregts, Marieke Haalboom, Reinoud P. H. Bokkers, Jean-Paul P. M. de Vries

**Affiliations:** 1 Department of Surgery, Division of Vascular Surgery, University Medical Center Groningen, University of Groningen, BA60, Hanzeplein 1, 9713 GZ Groningen, The Netherlands; 2 Department of Surgery, Division of Trauma Surgery, University Medical Center Groningen, University of Groningen, Groningen, The Netherlands; 3 Medical School Twente, Medisch Spectrum Twente, Enschede, The Netherlands; 4 Department of Radiology, Medical Imaging Center, University Medical Center Groningen, University of Groningen, Groningen, The Netherlands

**Keywords:** Tissue perfusion, Hyperspectral imaging, Transcutaneous oxygen pressure, Reproducibility of Results, Lower extremity, Tissue oxygenation

## Abstract

Purpose: Hyperspectral imaging (HSI) is a noninvasive spectroscopy technique for determining superficial tissue oxygenation. The HyperView™ system is a hand-held camera that enables perfusion image acquisition. The evaluation of superficial tissue oxygenation is warranted in the evaluation of patients with peripheral arterial disease. The aim was to determine the reliability of repeated HSI measurements. Methods: In this prospective cohort study, HSI was performed on 50 healthy volunteers with a mean age of 26.4 ± 2.5 years, at the lower extremity. Two independent observers performed HSI during two subsequent measurement sessions. Short term test–retest reliability and intra- and inter-observer reliability were determined, and generalizability and decision studies were performed. Transcutaneous oxygen pressure (TcPo_2_) measurements were also performed. Results: The short term test–retest reliability was good for the HSI values determined at the lower extremity, ranging from 0.72 to 0.90. Intra- and inter-observer reliability determined at different days were poor to moderate for both HSI (0.24 to 0.71 and 0.30 to 0.58, respectively) and TcPo_2_ (0.54 and 0.56, and 0.51 and 0.31, respectively). Reliability can be increased to >0.75 by averaging two measurements on different days. Conclusion: This study showed good short term test–retest reliability for HSI measurements, however low intra- and inter-observer reliability was observed for tissue oxygenation measurements with both HSI and TcPo_2_ performed at separate days in young healthy volunteers. Reliability of HSI can be improved when determined as a mean of two measurements taken on different days.

**Supplementary Information:**

The online version contains supplementary material available at 10.1007/s10877-021-00698-w.

## Introduction

Diagnostic techniques to determine perfusion and oxygenation in the lower extremities can potentially improve clinical decision making in patients with chronic limb threatening ischemia (CLTI) or patients with diabetic foot ulcers [1]. Early assessment of impaired tissue perfusion and/or oxygenation can be used for timely treatment to solve symptoms such as (rest) pain and non-healing foot ulcers. The current diagnostic techniques for detecting peripheral arterial occlusive disease (PAOD) focus mainly on detecting the presence of stenosis or occlusion in the large arteries but are not able to accurately determine tissue perfusion or oxygenation of the microvasculature. Adequate assessment of tissue perfusion and/or oxygenation at this level is essential for more accurate diagnosis, guidance during endovascular revascularization, and determination of sustained tissue perfusion increase during follow-up [1, 2].

Several techniques are currently available to determine tissue perfusion in the lower extremity. Transcutaneous oxygen pressure (TcPo_2_) measurement is the most commonly used technique for tissue perfusion [1, 3–8]. Unfortunately, this technique is time consuming, operator dependent, and the level of high-quality evidence remains low [2, 9, 10].

Hyperspectral imaging (HSI) is an emerging technique to evaluate localized superficial tissue oxygenation by measuring the concentrations of oxyhemoglobin (OxyHb) and deoxyhemoglobin (DeoxyHb). Visible light reflectance spectroscopy is used to determine these concentrations, which are presented in a color-coded image. The clinical use of this technique has been previously investigated in patients with cervical cancer and skin cancer as well as in an application for lower extremity perfusion measurements [11]. Previous studies determined superficial tissue oxygenation with HSI in patients with PAOD and diabetes mellitus (DM) and showed an association with severity of disease and wound healing [12–15]. Chin et al*.* showed that lower values of DeoxyHb were associated with peripheral arterial disease, and that DeoxyHb values correlated with ABI [16]. Contrary, an inverse relation have been described between HSI values and time to healing for foot ulcers [17]. Moreover, a correlation between ABI and HSI values was not observed in another study [18]. These results are, however, based on a small number of heterogeneous studies, and reliability and validity assessments of the measurements have not yet been performed.

HSI can be performed with commercially available devices that are floor-mounted systems consisting of a camera on a large workstation. Recently, HyperMed Inc. (Memphis, TN, USA) introduced a new HSI device called the HyperView™ system to determine the tissue oxygenation in the superficial layers of the skin. This system is a hand-held camera that enables measurement of regional tissue oxygenation at every desired location of the lower extremity. However, the operator dependency and measurement variation for the HyperView™ system are unknown.

This study investigated the reliability of the HyperView™ system for tissue oxygenation measurements of the lower extremity in healthy volunteers. HSI values were determined at different measurement locations at the lower extremity using a standardized measurement protocol. TcPo_2_ measurements were also performed as well as local skin temperature measurements. We have evaluated the short term test–retest reliability of HSI in addition to the intra- and inter-observer reliability of both HSI and TcPo_2_ measurements.

To incorporate several sources of variance in reliability assessment, a generalizability study was performed. The purpose of a generalizability study is to estimate variance components associated with the number of evaluations and observers. These variance components were then used for a decision study. This was performed to identify the optimal number of evaluations or observers in order to maximize reliability.

## Materials and methods

This single-center prospective cohort study included 50 healthy volunteers who were recruited among visitors and employees of the University Medical Center Groningen (UMCG). The study was conducted from June 2019 until November 2019. Inclusion criteria were palpable arterial pulses of the dorsalis pedis artery and posterior tibial artery and age > 18 years. The exclusion criteria were presence of previous or current PAOD, complaints of claudication, DM, neurologic diseases, any cardiovascular or pulmonary disease, recent trauma, and peripheral edema of the lower extremity. Demographics of the participants were recorded, including, sex, height, weight, age, and smoking status.

The Institutional Review Board reviewed the study (METc 2019/00,102) and determined the study does not fall under the scope of the Dutch Act on Medical Scientific Research Involving Human Beings (WMO). The study protocol was approved by the UMCG Central Ethics Review Board for non-WMO studies (register number: number #20,190,010). Study procedures were performed according to European privacy guidelines and according to the guidelines of the Declaration of Helsinki. Written informed consent was obtained from all participants. For privacy, all data were stored and analyzed after pseudo-anonymization.

### Measurement procedures

HSI was performed with the HyperView™ system to measure oxygenation of the superficial layers of the skin with a penetration depth of 1 mm to 2 mm. The measurements were performed according to a predefined measurement protocol designed to minimize possible variation in measurement conditions and performance [12]. For each measurement, participants were lying in semi-Fowler’s position, with 30° elevation of the bed, at least 5 min before imaging. The legs and feet were fixed in a foot rest, supported by pillows to ensure a comfortable position without pressure points without risk of movement between measurements. The HyperView^™^ camera was fixed on a tripod perpendicular to the skin to ensure stable and constant camera placement. Measurements were performed in the same room where room lighting was held constant during all measurements, and the ambient room temperature was maintained at 20 °C to 22 °C. Blood pressure was measured at the end of each measurement session.

Participants underwent HSI during four different measurement sessions at different days. To determine short term test–retest reliability, two consecutive images were taken during each session at every measurement location. To determine intra- and inter-observer reliability, the measurement sessions were performed by two independent observers. The measurements of the first two sessions were performed by the first observer, and the measurements of the third and fourth session were performed by the second observer. HSI was performed at both legs at the plantar side of the forefoot and at the lateral side of the calf muscle. The location at the lateral side of the calf was marked at 5 cm distally from the fibular head. Local skin temperature was recorded with an infrared thermography camera (FLIR Systems, Wilsonville, OR, USA).

The TcPo_2_ measurements were performed once during each session at the lateral side of the calf muscle of both legs, at the same location where HSI was performed. Performing TcPo_2_ at the plantar side of the foot was not feasible because of skin thickness and because of impossible probe attachment on the plantar side when the foot was in supine position. The TcPo_2_ measurements were performed with the Precisé 8001 (Medicap Homecare GmbH, Ulrichstein, Germany), a photo-optic measurement system. The marked location at the lateral side of the calf muscle was shaved before the session. The skin was disinfected, and the adhesive ring was applied with a drop of contact fluid. The TcPo_2_ sensor was attached, heated to the standard of 44 °C, and measurements were performed for at least 8 min until the oxygen partial pressure reached equilibrium [19].

### Hyperspectral image analysis

Levels of OxyHb and DeoxyHb and oxygen saturation were determined from the images using the software provided by HyperMed Inc. (version 1.2.2.) on the HyperView™ system. Regions of interest (ROI) with a diameter of 16 mm were manually placed at the caput of the third metatarsal at the plantar side of the foot and 5 cm distally from the fibular head at the lateral side of the calf, respectively, on the camera for every image.

### Statistical analysis

Data were collected in an online database using REDCap (Vanderbilt University, Nashville, TN, USA). Statistical analyses were performed with SPSS 23 software (IBM Corp, Armonk, NY, USA). Descriptive statistics are presented as mean ± standard deviation for normally distributed data or as medians with 25th and 75th percentiles otherwise. Differences between the measurement locations were calculated with a paired t test for normally distributed data or the Wilcoxon signed rank test otherwise. A Pearson correlation test was performed in one measurement session to determine the possible correlation between the TcPo_2_ and HSI values measured at the right and left calf. The short term test–retest reliability between consecutive images was determined using intraclass correlation coefficients (ICCs) and their 95% confidence intervals (CIs) based on an absolute agreement, two-way mixed model.

**Fig. 1:**
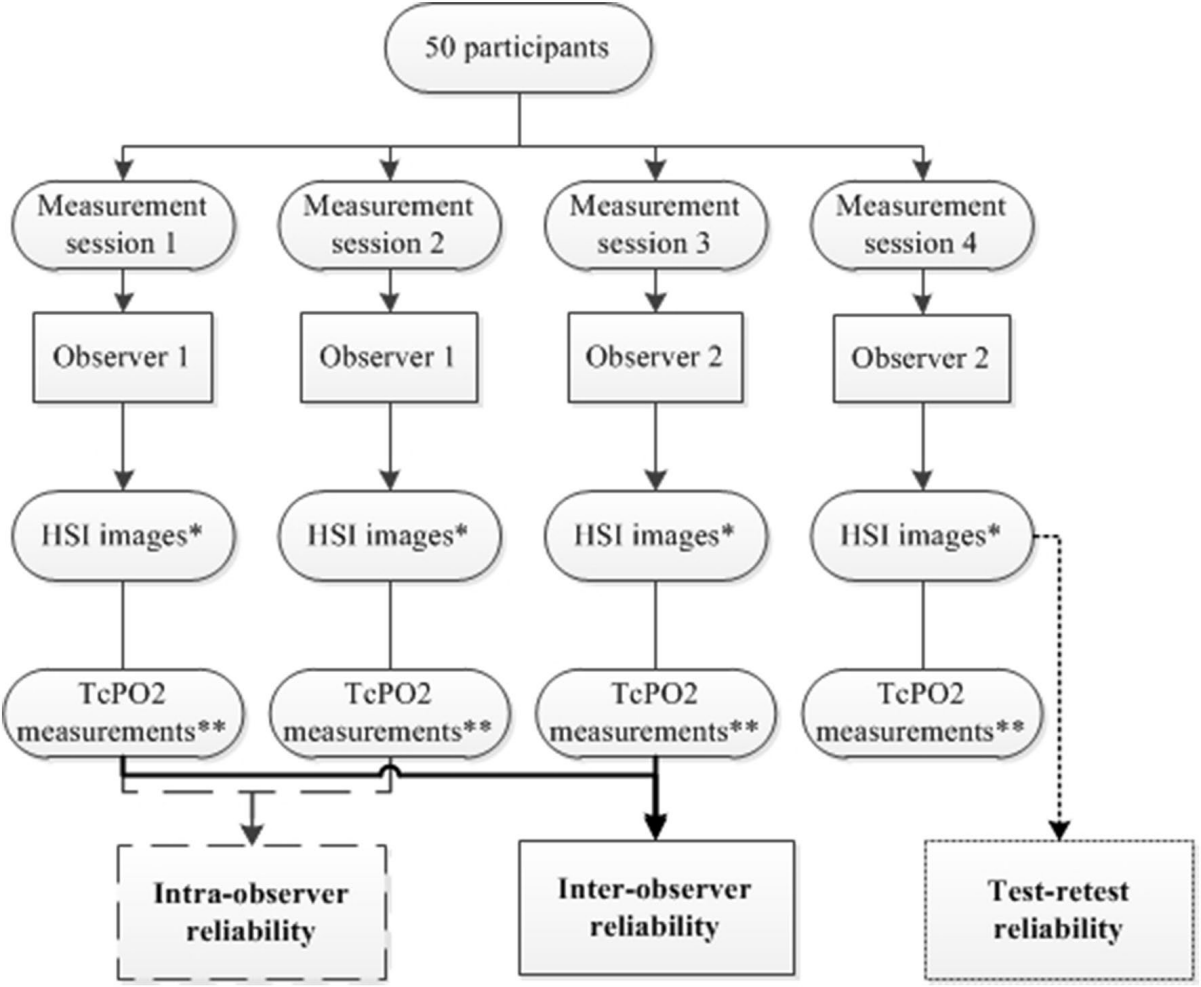
A flowchart of study procedures demonstrating the measurements used for the test–retest reliability and intra- and inter-observer reliability, respectively. During the measurement session, two consecutive images at each location were taken according to the standardized measurement protocol. The two consecutive images from one measurement session were used to determine test–retest reliability. The first image from measurement session 1 and 2 were used to determine intra-observer reliability. The first image from measurement session 1and 3 were used to determine inter-observer reliability. The same measurement sessions were used to determine the intra- and inter-observer reliability of TcPo_2_ measurements. * 2 images per measurement location; plantar side right foot, right calf, plantar side left foot, left calf. ** TcPo_2_ of the left and right calf

Intra-observer reliability was determined using ICCs based on a consistency, two-way random model. Inter-observer reliability was determined with an ICC based on an absolute agreement, two-way random model. ICC values of less than 0.5 indicated poor reliability, values between 0.5 and 0.75 indicated moderate reliability, values between 0.75 and 0.9 were considered good reliability, and values greater than 0.9 reflected excellent reliability [20]. A flowchart of study procedures and reliability assessment is shown in Fig. 1. Measurement error was determined for short term test–retest, intra- and inter-observer reliability with the standard error of measurement (SEM), limits of agreement, smallest detectable change, and Bland–Altman plots [21, 22]. The limits of agreement were calculated with the mean difference between two measurements ± 1.96⋅SD of the mean difference. The smallest detectable change was calculated as 1.96⋅√2⋅SEM.

A generalizability study was performed to determine intra- and inter-observer reliability considering measurement sessions (on different days) and observers as independent sources of variation [21]. In the generalizability and decision study we theoretically added different numbers of observers and evaluations as a strategy to determine improvement of reliability. Generalizability (G) coefficients for consistency, based on a two-facet fully crossed design, were calculated with variance components obtained through analysis of variance (restricted maximum likelihood). A decision study was performed to determine the most efficient strategy to increase reliability. Two possible strategies are averaging multiple measurements on different days by the same observer or averaging the measurements from different observers on different days. Therefore, in the decision study, G coefficients for intra-observer and inter-observer reliability based on consistency were determined when averaging the variance of observers and measurements sessions [23].

## Results

Of the 50 healthy volunteers, 29 were men, the mean age was 26.4 ± 2.5 years, the mean systolic blood pressure was 121 ± 11 mmHg, the mean diastolic blood pressure was 75 ± 7 mmHg, and the median body mass index was 22 (20.5–23.5) kg/m^2^. Two of the healthy volunteers were smokers. The median duration of one measurement session was 41.0 (39.0–43.0) minutes. The oxygenation values, TcPo_2_ measurements, and local skin temperature, and differences between measurement locations are presented in Table 1.

**Table 1:** Hyperspectral imaging values, TcPo_2_ and local skin temperature of 50 healthy volunteers at different locations of the lower leg

	Right foot	Left foot	Right vs. Left foot (*p* value)	Right calf	Left calf	Right vs. Left calf (*p* value)
OxyHb (a.u.)	85.0 ± 25.9^*^	78.2 ± 23.7^*^	.003	38.9 ± 10.5^*^	42.9 ± 12.1^*^	.001
DeoxyHb (a.u.)	64.9 ± 14.3^*^	71.3 ± 15.4^*^	<.001	42.2 ± 11.6^*^	40.7 ± 11.2^*^	.205
Oxygen saturation (%)	55.8 ± 10.9^*^	51.6 ± 10.8	<.001	48.2 ± 10.7^*^	51.2 ± 10.4	.008
TcPo_2_ (mm Hg)	…	…		66.7 ± 13.5	61.1 ± 11.3	<.001
Skin temperature (°C)	27.6 ± 2.7^*^	27.7 ± 2.8^*^	.879	32.7 ± 0.8^*^	32.4 ± 0.9^*^	<.001

HSI values are the mean from the first image from one measurement session of 50 participants. Values are reported as mean ± standard deviation. *a.u.* arbitrary units, *TcPo*_*2*_ transcutaneous oxygen pressure. ^*^Statistically significant difference between foot and calf within the ipsilateral leg *P* < .001

Nearly all tissue oxygenation measurements were significantly different between the locations on the feet and calves and between the right and left feet or calves. Only skin temperature was not different between the right and left feet, DeoxyHb was not significantly different between the right and left calves, and oxygen saturation was not different between the left feet and calves.

OxyHb correlated significantly with TcPo_2_ at the right calf (R = −0.279, *p* < 0.05), but not at the left calf (R = −0.214, *p* = 0.140). DeoxyHb and oxygen saturation did not correlate with TcPo_2_ measurements.

### Short term test–retest reliability

The short term test–retest reliability for the HSI values is shown in Table 2. The ICCs (95% CI) for the oxygenation values ranged from 0.72 (0.56–0.83) to 0.90 (0.83–0.94), which indicated good reliability. The DeoxyHb value showed the highest reliability for every measurement location according to small measurement error and high ICC. The OxyHb value at the plantar side of the right and left feet showed the highest measurement errors of 9.6 and 10.1, respectively. Measurement error seemed smaller for oxygenation levels measured at the calves compared to the feet. Bland–Altman plots of short term test–retest agreement showed lower agreement for OxyHb measurements at the feet (Fig. S1). There was no systematic error for OxyHb and for DeoxyHb measured at the feet, and only a small error for DeoxyHb at the right calves (Fig. S1).

**Table 2:** Short term test–retest reliability and measurement error of two consecutive hyperspectral images taken at one measurement session

Location		OxyHb (a.u.)	DeoxyHb (a.u.)	Oxygen saturation (%)
Right foot	SEM_agree_	10.1	4.9	4.3
	LoA	(−28.3 to 28.4)	(− 4.0 to 13.7)	(−12.2 to 12.0)
	SDC	28.1	13.7	11.6
	ICC_agree_	0.85	0.89	0.84
	95% CI	(0.75–0.91)	(0.82–0.94)	(0.73–0.91)
Right calf	SEM_agree_	3.5	3.7	3.8
	LoA	(−9.8 to 10.0)	(−11.5 to 8.4)	(− 9.5 to 11.3)
	SDC	9.8	10.3	10.5
	ICC_agree_	0.89	0.90	0.88
	95% CI	(0.81–0.94)	(0.83–0.94)	(0.80–0.93)
Left foot	SEM_agree_	9.6	6.0	5.2
	LoA	(−28.0 to 25.2)	(−13.9 to 18.4)	(−15.6 to 12.7)
	SDC	26.5	16.6	14.3
	ICC_agree_	0.82	0.84	0.75
	95% CI	(0.71–0.89)	(0.74–0.91)	(0.61–0.85)
Left calf	SEM_agree_	5.3	4.3	5.3
	LoA	(−13.4 to 15.7)	(−13.0 to 10.6)	(− 12.8 to 16.0)
	SDC	14.6	11.9	14.6
	ICC_agree_	0.82	0.84	0.72
	95% CI	(0.70–0.89)	(0.73–0.91)	(0.56–0.83)

Values are determined with the two subsequent images from one measurement session for 50 participants. *SEM*_*agree*_ standard error of measurement for absolute agreement, *LoA* limits of agreement, *ICC*_*agree*_ Intraclass correlation for absolute agreement, *95% CI* 95% Confidence intervals for the ICC, *SDC* smallest detectable change

### Intra-observer reliability

The intra-observer reliability at different days for the HSI values and TcPo_2_ measurements is shown in Table 3. The ICCs for the oxygenation values ranged from 0.24 (−0.04–0.49) to 0.71(0.54–0.82), indicating poor to moderate reliability. The DeoxyHb value showed the highest reliability, with ICCs ranging from 0.57 (0.34–0.73) to 0.71 (0.54–0.82), indicating moderate reliability. The OxyHb values showed the highest measurement error (21.2 and 17.3, respectively) and the lowest ICC (0.24 (−0.04–0.49) and 0.41 (0.16–0.62), respectively) at the right and left plantar side of the feet. Overall measurement error seemed smaller at the calves compared with the feet. Bland–Altman plots of intra-observer agreement showed low agreement for OxyHb measurements at the feet (Fig. S2). The plots showed no systematic error for OxyHb at the feet and calves, and there was a small error for DeoxyHb measured at the right and left feet and left calf (Fig. S2). ICCs of TcPo_2_ measurements at the right and left leg were 0.54 (0.49–0.59) and 0.56 (0.52–0.60), respectively, indicating moderate reliability. Bland–Altman plots of agreement for TcPo_2_ measurements showed no systematic error (Fig. S3).

**Table 3:** Intra-observer reliability and measurement error for HSI at two different measurement sessions performed by one observer together with TcPo_2_ measurements from these sessions

Location		OxyHb (a.u.)	DeoxyHb (a.u.)	Oxygen saturation (%)	TcPo_2_ (mm Hg)
Right foot	SEM_cons_	21.2	8.7	8.6	
	LoA	(−59.5 to 57.9)	(−29.3 to 19.4)	(−22.1 to 25.5)	
	SDC	58.7	24.3	23.8	
	ICC_cons_	0.24	0.71	0.33	
	95% CI	(−0.04–0.49)	(0.54–0.82)	(0.06–0.56)	
Right calf	SEM_cons_	6.1	6.9	6.9	9.1
	LoA	(−19.0 to 14.9)	(−20.3 to 18.1)	(−19.7 to 18.8)	(−25.0 to 25.7)
	SDC	16.9	19.2	19.2	25.1
	ICC_cons_	0.62	0.59	0.57	0.54
	95% CI	(0.42–0.77)	(0.38–0.75)	(0.34–0.73)	(0.49–0.59)
Left foot	SEM_cons_	17.3	10.0	7.8	
	LoA	(−50.3 to 45.7)	(−33.6 to 21.8)	(−20.0 to 23.1)	
	SDC	48.0	27.7	21.6	
	ICC_cons_	0.41	0.59	0.37	
	95% CI	(0.16–0.62)	(0.37–0.74)	(0.11–0.59)	
Left calf	SEM_cons_	7.4	8.2	8.8	6.9
	LoA	(−19.2 to 21.6)	(−26.0 to 19.2)	(−21.7 to 27.2)	(− 19.2 to 19.8)
	SDC	20.4	22.6	24.5	19.2
	ICC_cons_	0.46	0.57	0.44	0.56
	95% CI	(0.20–0.65)	(0.34–0.73)	(0.18–0.64)	(0.52–0.60)

Values are determined with the first image from the first session with the first image from the second session for 50 participants. *SEM*_*cons*_ standard error of measurement for consistency, *LoA* limits of agreement, *ICC*_*cons*_ Intraclass correlation for consistency, *95% CI* 95% Confidence intervals for the ICC, *TcPo*_*2*_ transcutaneous oxygen pressure, *SDC* smallest detectable change

### Inter-observer reliability

The inter-observer reliability at different days for the HSI values and TcPo_2_ measurements is shown in Table 4. The ICCs ranged from 0.30 (0.03–0.53) to 0.58 (0.36–0.74), indicating poor to moderate reliability. The OxyHb value measured at the plantar side of right and left the feet showed the highest measurement error (15.9 and 18.0, respectively). The OxyHb and DeoxyHb values appear to have a higher measurement error at the feet compared to the calves. The Bland–Altman plot of inter-observer reliability showed low agreement of OxyHb measurements at the feet (Fig. S4). The plots showed a systematic error for DeoxyHb measured at the right and left feet and the left calf (Fig. S4). ICCs of TcPo_2_ measurements at the right and left leg were 0.52 (0.47–0.58) and 0.31 (0.21–0.45), respectively, indicating poor and moderate reliability. Bland–Altman plots of agreement for TcPo_2_ measurements showed no systematic error (Fig. S3).

**Table 4:** Inter-observer reliability and measurement error for HSI at two different measurement sessions performed by two observers together with TcPo_2_ measurements from these sessions

Location		OxyHb (a.u.)	DeoxyHb (a.u.)	Oxygen saturation (%)	TcPo_2_ (mm Hg)
Right foot	SEM_agree_	15.9	12.0	8.1	
	LoA	(−39.2 to 48.3)	(−37.1 to 16.7)	(−14.2 to 25.5)	
	SDC	44.2	33.3	22.6	
	ICC_agree_	0.58	0.43	0.45	
	95% CI	(0.36–0.74)	(0.07–0.67)	(0.16–0.66)	
Right calf	SEM_agree_	8.0	9.0	9.1	9.7
	LoA	(−22.3 to 22.4)	(−26.4 to 23.8)	(–24.7 to 26.4)	(–24.6 to 29.1)
	SDC	22.1	25.0	25.3	26.8
	ICC_agree_	0.33	0.32	0.26	0.52
	95% CI	(0.05–0.55)	(0.05–0.55)	(–0.30 to 0.50)	(0.47–0.58)
Left foot	SEM_agree_	18.0	11.1	8.3	
	LoA	(–46.8 to 53.4)	(–34.5 to 16.5)	(–17.1 to 26.2)	
	SDC	50.0	30.8	23.2	
	ICC_agree_	0.30	0.48	0.30	
	95% CI	(0.03–0.53)	(0.13–0.70)	(0.05–0.53)	
Left calf	SEM_agree_	6.9	7.6	8.5	10.0
	LoA	(–15.8 to 21.4)	(–23.8 to 13.4)	(−16.6 to 26.7)	(−23.5 to 31.3)
	SDC	19.2	21.1	23.6	27.6
	ICC_agree_	0.46	0.57	0.42	0.31
	95% CI	(0.22–0.65)	0.28–0.75)	(0.15–0.62)	(0.21–0.45)

Values are determined with the first image from measurement session #1 performed by observer #1 and the first image from measurement session #3 performed by observer #2 for 50 participants. *SEM*_*agree*_ standard error of measurement for agreement, *LoA* limits of agreement, *ICC*_*agree*_ Intra Class Correlation for absolute agreement, *95% CI* 95% Confidence intervals for the ICC, *TcPo*_*2*_ transcutaneous oxygen pressure, *SDC* smallest detectable change

### Generalizability and decision study

Generalizability coefficients of intra-observer reliability based on consistency for measurements on different days are shown in Table 5. The intra-observer generalizability coefficients of HSI (marked with *) ranged from 0.35 to 0.69 when all measurements of all participants performed by the same observer were compared, indicating poor to moderate reliability. In a situation where two repeated measurements were performed by one observer on different days, a G coefficient of 0.46 to 0.71 was achieved for OxyHb and >0.75 for DeoxyHb, indicating moderate to good agreement. When the repeated measurements were increased to four by one observer, the G coefficient increased from 0.52 to 0.83 for OxyHb and >0.85 for DeoxyHb.

**Table 5:** Intra-observer reliability based on generalizability and decision studies of HSI measurements

	OxyHb				DeoxyHb				Oxygen saturation			
Right foot												
*n*_m↓_*n*_o→_	1	2	4	6	1	2	4	6	1	2	4	6
1	0.40^*^	0.52	0.62	0.67	0.65^*^	**0.75**	**0.83**	**0.87**	0.38^*^	0.43	0.49	0.51
2	0.57	0.68	**0.77**	**0.80**	**0.79**	**0.86**	**0.91**	**0.93**	0.55	0.61	0.65	0.68
4	0.73	**0.81**	**0.87**	**0.89**	**0.88**	**0.92**	**0.95**	**0.96**	0.71	**0.75**	**0.79**	**0.81**
6	**0.80**	**0.87**	**0.91**	**0.92**	**0.92**	**0.95**	**0.97**	**0.98**	**0.79**	**0.82**	**0.85**	**0.86**
Left foot												
*n*_m↓_*n*_o→_	1	2	4	6	1	2	4	6	1	2	4	6
1	0.40^*^	0.49	0.58	0.63	0.60^*^	0.72	**0.82**	**0.85**	0.35^*^	0.43	0.51	0.55
2	0.57	0.66	0.74	**0.77**	**0.75**	**0.84**	**0.90**	**0.92**	0.52	0.60	0.67	0.71
4	0.73	**0.79**	**0.85**	**0.87**	**0.85**	**0.91**	**0.95**	**0.96**	0.68	**0.75**	**0.80**	**0.83**
6	**0.80**	**0.85**	**0.89**	**0.91**	**0.90**	**0.94**	**0.96**	**0.97**	**0.77**	**0.82**	**0.86**	**0.88**
Right calf												
*n*_m↓_*n*_o→_	1	2	4	6	1	2	4	6	1	2	4	6
1	0.37^*^	0.54	0.70	**0.78**	0.65^*^	**0.76**	**0.85**	**0.89**	0.65^*^	**0.76**	**0.85**	**0.89**
2	0.46	0.63	**0.77**	**0.83**	**0.79**	**0.86**	**0.92**	**0.94**	**0.79**	**0.86**	**0.92**	**0.94**
4	0.52	0.68	**0.81**	**0.87**	**0.88**	**0.93**	**0.96**	**0.97**	**0.88**	**0.93**	**0.96**	**0.97**
6	0.55	0.71	**0.83**	**0.88**	**0.92**	**0.95**	**0.97**	**0.98**	**0.92**	**0.95**	**0.97**	**0.98**
Left calf												
*n*_m↓_*n*_o→_	1	2	4	6	1	2	4	6	1	2	4	6
1	0.55^*^	0.71	**0.83**	**0.88**	0.69^*^	**0.80**	**0.89**	**0.92**	0.57^*^	0.71	**0.82**	**0.87**
2	0.71	**0.83**	**0.91**	**0.94**	**0.82**	**0.89**	**0.94**	**0.96**	0.73	**0.83**	**0.90**	**0.93**
4	**0.83**	**0.91**	**0.95**	**0.97**	**0.90**	**0.94**	**0.97**	**0.98**	**0.84**	**0.91**	**0.95**	**0.97**
6	**0.88**	**0.94**	**0.97**	**0.98**	**0.93**	**0.96**	**0.98**	**0.99**	**0.89**	**0.94**	**0.97**	**0.98**

*HSI* hyperspectral imaging*, n*_m_ alternative number of measurements, *n*_o_ alternative number of observers. ^*^generalizability coefficients determined with all measurements performed by the same observer. Coefficients in bold exceed 0.75, indicating good reliability. *OxyHb* Oxyhemoglobin, *DeoxyHb* deoxyhemoglobin

Generalizability coefficients based on inter-observer reliability for measurements on different days are shown in Table 6. The inter-observer generalizability coefficients of HSI (marked with *) ranged from 0.27 to 0.59 when all measurements of all participants performed by different observers were compared, indicating poor to moderate reliability. In a situation where we increased the repeated measures or observers to two, the reliability of the measurements increased, and precision was again better for DeoxyHb than for OxyHb or oxygen saturation. When two observers performed two measurements, better overall precision was reached, with OxyHb from 0.52 to 0.83 and DeoxyHb from 0.71 to 0.83.

**Table 6:** Inter-observer reliability based on generalizability and decision studies of HSI measurements

	OxyHb				DeoxyHb				Oxygen saturation			
Right foot												
*n*_m↓_*n*_o→_	1	2	4	6	1	2	4	6	1	2	4	6
1	0.42^*^	0.59	0.74	**0.81**	0.53^*^	0.69	**0.82**	**0.87**	0.36^*^	0.52	0.69	**0.77**
2	0.54	0.70	**0.82**	**0.87**	0.62	**0.77**	**0.87**	**0.91**	0.41	0.58	0.73	**0.81**
4	0.64	**0.78**	**0.88**	**0.92**	0.69	**0.82**	**0.90**	**0.93**	0.46	0.63	**0.77**	**0.84**
6	0.70	**0.82**	**0.90**	**0.93**	0.72	**0.83**	**0.91**	**0.94**	0.48	0.65	**0.79**	**0.85**
Left foot												
*n*_m↓_*n*_o→_	1	2	4	6	1	2	4	6	1	2	4	6
1	0.27^*^	0.43	0.60	0.69	0.59^*^	0.74	**0.85**	**0.89**	0.31^*^	0.48	0.65	**0.73**
2	0.35	0.52	0.68	**0.76**	0.71	**0.83**	**0.91**	**0.94**	0.39	0.56	0.72	**0.79**
4	0.42	0.59	0.74	**0.81**	**0.80**	**0.89**	**0.94**	**0.96**	0.46	0.63	**0.77**	**0.84**
6	0.45	0.62	**0.77**	**0.83**	**0.84**	**0.91**	**0.95**	**0.97**	0.50	0.66	**0.80**	**0.86**
Right calf												
*n*_m↓_*n*_o→_	1	2	4	6	1	2	4	6	1	2	4	6
1	0.48^*^	0.65	**0.79**	**0.85**	0.45^*^	0.62	**0.77**	**0.83**	0.39^*^	0.56	0.72	**0.79**
2	0.65	**0.79**	**0.88**	**0.92**	0.55	0.71	**0.83**	**0.88**	0.48	0.65	**0.79**	**0.85**
4	**0.79**	**0.88**	**0.94**	**0.96**	0.61	**0.76**	**0.86**	**0.90**	0.54	**0.70**	**0.82**	**0.88**
6	**0.85**	**0.92**	**0.96**	**0.97**	0.64	**0.78**	**0.87**	**0.91**	0.56	**0.72**	**0.84**	**0.89**
Left calf												
*n*_m↓_*n*_o→_	1	2	4	6	1	2	4	6	1	2	4	6
1	0.55^*^	0.71	**0.83**	**0.88**	0.59^*^	0.74	**0.85**	**0.90**	0.48^*^	0.65	**0.79**	**0.85**
2	0.71	**0.83**	**0.91**	**0.94**	0.70	**0.82**	**0.90**	**0.93**	0.61	**0.76**	**0.86**	**0.90**
4	**0.83**	**0.91**	**0.95**	**0.97**	**0.77**	**0.87**	**0.93**	**0.95**	0.70	**0.83**	**0.90**	**0.93**
6	**0.88**	**0.94**	**0.97**	**0.98**	**0.80**	**0.89**	**0.94**	**0.96**	0.74	**0.85**	**0.92**	**0.95**

*HSI* hyperspectral imaging*, n*_m_ alternative number of measurements, *n*_o_ alternative number of observers. ^*^Generalizability coefficients determined with all measurements performed by different observers. Coefficients in bold exceed 0.75, indicating good reliability. *OxyHb* Oxyhemoglobin, *DeoxyHb* Deoxyhemoglobin

## Discussion

The portable HyperView™ system is a hand-held and user-friendly HSI system that can be used to quantify superficial tissue oxygenation of the feet and calf with good short term test–retest reliability. Intra- and inter-observer reliability of HSI was poor to moderate and for TcPo_2_ measurements alike. Reliability of HSI could be improved when determined as a mean of two measurements taken on different days. The OxyHb, DeoxyHb, and oxygenation values were comparable with a previous study of HSI in healthy volunteers [21, 24]. OxyHb and DeoxyHb values were significantly higher at the plantar side of the foot compared with the calf, which can potentially be explained by differences in texture of skin, color, thickness, and lack of hair follicles. Also, the perfusion of the plantar side of the foot is supplied by the largest number of angiosomes [25]. Skin temperature can also influence the superficial tissue oxygenation. The skin temperature was lower at the plantar side of the foot with a greater dispersion compared with the calf measurements. Local skin temperature and therefore oxygenation values may thus be more variable at the feet.

Reliability and validity data of the HyperView™ system are a prerequisite before it can be implemented into clinical practice. The short term test–retest results in our study show high degree of agreement and low measurement error in superficial tissue oxygenation values between two consecutive images taken 2 min apart. These measurements were performed following a predefined standardized measurement protocol [12]. Clinical measurements without such a strict protocol may vary more. The short term test–retest reliability was similar to previous findings in a study with patients with PAOD, where HSI was performed with a non-hand-held device from the same manufacturer [26]. The DeoxyHb value showed lower SEMs and higher ICCs compared with OxyHb for both feet and calves. This is in line with the intra- and inter-observer reliability found in HSI measurements in patients with PAOD [26].

This is the first study to investigate the reliability of tissue oxygenation measurements on different days. The observed reliability is not significantly different for the right and left side of the calf and the foot. The observed differences in skin oxygenation are likely due to chance, as structural differences between the right and left sides are not expected. For measurements performed at different days, there was a low intra- and inter-observer reliability. Neville et al. performed repeated HSI in healthy volunteers 8 h apart, although reliability was not determined, and they showed no statistically significant differences between the measurements [24]. The intra- and inter-observer reliability for TcPo_2_ measurements on different days was poor to moderate and similar to the HSI measurements at the calf. We therefore hypothesize that superficial tissue oxygenation values vary significantly in healthy volunteers on different days, explaining the low reliability of both the HSI and TcPo_2_ measurements. The reason for the low reliability and agreement might be caused by measurement error or by a high variability in superficial tissue oxygenation caused by many possible sources of variation. To better assess the reliability taking into account different days and observers as sources of variation in this study design of repeated measurement sessions, the generalizability study showed moderate intra-observer reliability for DeoxyHb at every location. These values are higher than the previously determined ICCs, but were obtained in a more realistic manner by taking into account the variance caused by observers and measurement sessions.

What needs to be considered is that variations in measurements can partly be explained by variable factors such as physical activity, stress, smoking, caffeine intake, difference in environmental temperature before and during measurements, vasoconstriction, skin color changes, and skin temperature. All of these factors can be different between participants, which is shown in the large standard deviation of HSI values determined at one moment (Table 2) and can be different within the participants, which results in a low reliability score at different measurement moments (Tables 4 and 5). These sources of variation are difficult to standardize during measurements. However, these sources of variation will also be present when performing measurements in patients with PAOD. A possible solution to interpret the results of HSI can therefore be to average measurements on different days and observing trends during consecutive measurements at different days.

The G coefficients obtained from the decision study suggest that the reliability of the measurements is increased by averaging multiple measurements obtained over different days. Two repeated measurements are sufficient to obtain overall good reliability for DeoxyHb, but four to six measurements are required to obtain reliable measurements of OxyHb. Averaging values from multiple measurements and observing general trends rather than using a strict cutoff on a single measurement should be considered to accurately determine lower extremity perfusion. For clinical implementation, monitoring of superficial tissue oxygenation should be performed with an average of at least two measurements.

TcPo_2_ measurements were also performed to determine tissue perfusion [8]. The mean TcPo_2_ values exceeded the minimal 50 mm Hg, which is considered normal in healthy subjects [7]. The ICC of TcPo_2_ measured at different days was low, underlining the large variation in normal tissue perfusion in healthy participants on different days. The low reliability can also be a result of measurement error of the TcPo_2_ measurements, however a short term test–retest reliability was not performed for these measurements. This renders it uncertain whether the low reliability arises mainly from the variation in superficial tissue oxygenation, as it does in HSI measurements. The reliability of TcPo_2_ at different days has been studied to limited extent. De Graaff et al. showed similar moderate reliability and high measurement error [27]. However, one other previous study measuring TcPo_2_ during exercise on different days showed good reliability [28]. The use of a provocation test, such as exercise or cuff occlusion, may lead to a better reliability of measurements for HSI as well.

The lack of a provocation test to induce changes in lower limb perfusion is one of the limitations in this study. Another limitation is the lack of test–retest reliability for the TcPo_2_ measurements as only one TcPo_2_ measurement has been performed. Previous studies however showed good test–retest reliability for TcPo_2_ [27]. Besides this, participants had only 5 min of rest before measurements, which might not be enough to attain resting state perfusion for every participant depending on their level of physical activity before the study, this may have introduced a variation between participants. Another limitation of the study is that the time intervals between measurement sessions could not be standardized because of availability of the research rooms, the research physicians and the volunteers. Moreover, this study is limited to HSI measurements in healthy volunteers, and cannot be extrapolated to patients. The variability of superficial tissue oxygenation might differ between healthy tissue and tissue from patients suffering PAOD or DM. The low reliability coefficients in this study cannot directly be translated to a patient group. Notwithstanding a large variation in HSI values, these may be significantly lower in patients groups as has been shown in studies using other HSI devices [12]. It should be taken into consideration that this study used optimal measurement conditions, the lack of which could result in lower reliability in clinical practice.

In future research dedicated to HSI measurements with the HyperView^™^ system, the validity of measurements should be determined in patients with PAOD and DM. This should be compared with TcPo_2_ measurements as well as with conventional diagnostics like ankle brachial indices and Doppler ultrasound. In addition, it is important to investigate whether HSI can differentiate tissue oxygenation between the respective angiosomes in the lower extremity of patients.

## Conclusions

To conclude, this study showed good short term test–retest reliability, but low intra- and inter-observer reliability for superficial tissue oxygenation measurements with both HSI and TcPo_2_ performed on separate days in healthy volunteers. The use of the average of two measurements taken on different days would be a possible solution to increase the reliability of HSI. The current results may not be representative for patients with impaired tissue perfusion. Reliability and validity should thus also be determined in PAOD and DM patients before HSI can be implemented in clinical practice.

## Supplementary Information

Below is the link to the electronic supplementary material.

Supplementary file1 (DOCX 15 kb)

Supplementary file2 (TIFF 725 kb)

Supplementary file3 (TIFF 499 kb)

Supplementary file4 (TIFF 783 kb)

Supplementary file5 (TIFF 870 kb)

## Data Availability

Due to the nature of this research, participants of this study did not agree for their data to be shared publicly, so supporting data is not available.

## References

[1] Misra S, Shishehbor MH, Takahashi EA, Aronow HD, Brewster LP, Bunte MC (2019). Perfusion assessment in critical limb ischemia: Principles for understanding and the development of evidence and evaluation of devices: A scientific statement from the American Heart Association. Circulation.

[2] Rogers RK, Montero-Baker M, Biswas M, Morrison J, Braun J (2020). Assessment of foot perfusion: Overview of modalities, review of evidence, and identification of evidence gaps. Vasc Med (United Kingdom).

[3] Ma KF, Kleiss SF, Schuurmann RCL, Bokkers RPH, Ünlü Ç, De Vries J-PPM (2019). A systematic review of diagnostic techniques to determine tissue perfusion in patients with peripheral arterial disease. Expert Rev Med Devices.

[4] Marcoccia A, Klein-Weigel PF, Gschwandtner ME, Wautrecht JC, Matuska J, Rother U (2020). Microcirculatory assessment of vascular diseases. Vasa - Eur J Vasc Med.

[5] Scheffler A, Rieger H (1992). A comparative analysis of transcutaneous oximetry (tcPO2) during oxygen inhalation and leg dependency in severe peripheral arterial occlusive disease. J Vasc Surg United States.

[6] Scheffler A, Rieger H (1992). Clinical information content of transcutaneous oxymetry (tcpO2) in peripheral arterial occlusive disease (a review of the methodological and clinical literature with a special reference to critical limb ischaemia). Vasa Switzerland.

[7] Fife CE, Smart DR, Sheffield PJ, Hopf HW, Hawkins G, Clarke D (2009). Transcutaneous oximetry in clinical practice: Consensus statements from an expert panel based on evidence. Undersea Hyperb Med.

[8] Sheffield PJ (1998). Measuring tissue oxygen tension: a review. Undersea Hyperb Med J Undersea Hyperb Med Soc Inc United States.

[9] Wang Z, Hasan R, Firwana B, Elraiyah T, Tsapas A, Prokop L (2016). A systematic review and meta-analysis of tests to predict wound healing in diabetic foot. J Vasc Surg Elsevier.

[10] Wütschert R, Bounameaux H (1997). Determination of amputation level in ischemic limbs: Reappraisal of the measurement of TcPO2. Diabetes Care.

[11] Lu G, Fei B (2014). Medical hyperspectral imaging: A review. J Biomed Opt.

[12] Kleiss SF, Ma KF, Schuurmann RC, El Moumni M, Zeebregts CJ, Bokkers RP (2019). Hyperspectral imaging for noninvasive tissue perfusion measurements of the lower leg: Review of literature and introduction of a standardized measurement protocol with a portable system. J Cardiovasc Surg (Torino).

[13] Grambow E, Dau M, Sandkuhler NA, Leuchter M, Holmer A, Klar E (2019). Evaluation of peripheral artery disease with the TIVITA^®^ Tissue hyperspectral imaging camera system. Clin Hemorheol Microcirc.

[14] Sumpio BJ, Citoni G, Chin JA, Sumpio BE (2016). Use of hyperspectral imaging to assess endothelial dysfunction in peripheral arterial disease. J Vasc Surg..

[15] Greenman RL, Panasyuk S, Wang X, Lyons TE, Dinh T, Longoria L (2005). Early changes in the skin microcirculation and muscle metabolism of the diabetic foot. Lancet.

[16] Chin JA, Wang EC, Kibbe MR (2011). Evaluation of hyperspectral technology for assessing the presence and severity of peripheral artery disease. J Vasc Surg..

[17] Jeffcoate WJ, Clark DJ, Savic N, Rodmell PI, Hinchliffe RJ, Musgrove A (2015). Use of HSI to measure oxygen saturation in the lower limb and its correlation with healing of foot ulcers in diabetes. Diabet Med.

[18] Jafari-Saraf L, Gordon IL (2010). Hyperspectral imaging and ankle: Brachial indices in peripheral arterial disease. Ann Vasc Surg..

[19] Précise 8001 - User Manual. 1st ed. Ulrichstein: medicap homecare GmbH; 2016.

[20] Koo TK, Li MY (2016). A guideline of selecting and reporting intraclass correlation coefficients for reliability research. J Chiropr Med..

[21] de Vet HCW, Terwee CB, Mokkink LB, Knol DL. Reliability. Meas Med. Cambridge: Cambridge University Press; p. 96–149.

[22] de Vet HCW, Terwee CB, Knol DL, Bouter LM (2006). When to use agreement versus reliability measures. J Clin Epidemiol.

[23] Shavelson R, Webb N. Generalizability Theory. In: Green J, Camilli G, Elmore P, editors. Handb Complement methods Educ Res. Lawerence Erlbaum Associates Publishers; 2006. p. 5309–22.

[24] Neville R, Gupta S (2009). Establishment of normative perfusion values using hyperspectral tissue oxygenation mapping technology. Vasc Dis Manag.

[25] Attinger CE, Evans KK, Bulan E, Blume P, Cooper P (2006). Angiosomes of the foot and ankle and clinical implications for limb salvage: Reconstruction, incisions, and revascularization. Plast Reconstr Surg.

[26] Chiang N, Jain JK, Sleigh J, Vasudevan T (2017). Evaluation of hyperspectral imaging technology in patients with peripheral vascular disease. J Vasc Surg..

[27] De Graaff JC, Ubbink DT, Legemate DA, De Haan RJ, Jacobs MJHM (2001). Interobserver and intraobserver reproducibility of peripheral blood and oxygen pressure measurements in the assessment of lower extremity arterial disease. J Vasc Surg.

[28] Henni S, Semporé YW, Le Meliner T, Ouedraogo N, Hamel JF, Abraham P (2018). Intra-test and test-retest reliability of exercise oximetry in arterial claudication. Microvasc Res Elsevier.

