# Supplementary material for: Reliability assessment of hyperspectral imaging with the HyperView™ system for lower extremity superficial tissue oxygenation in young healthy volunteers
Source: J Clin Monit Comput. 2021 Apr 12;36(3):713–23. doi: 10.1007/s10877-021-00698-w (PMC9162963; doi:10.1007/s10877-021-00698-w)
Supplement: Supplementary file 1 — Supplementary file1 (DOCX 15 kb) [file 10877_2021_698_MOESM1_ESM.docx]

**Supplementary Information**

The following supplementary figures are available for this manuscript: **Figure S1.** Bland-Altman plots of the test-retest agreement for oxyhemoglobin (OxyHb) and deoxyhemoglobin (DeoxyHb) measurements. The center line represents the mean difference, and the dashed lines are the upper and lower limit of agreement. a.u.: arbitrary units. **Figure S2.** Bland-Altman plots of the intra-observer agreement for oxyhemoglobin (OxyHb) and deoxyhemoglobin (DeoxyHb) measurements. The center line represents the mean difference, and the dashed lines are the upper and lower limit of agreement. a.u.: arbitrary units. **Figure S3.** Bland-Altman plots of the intra-observer and inter-observer agreement for transcutaneous oxygen pressure measurements (TcPO2) measurements. The center line represents the mean difference, and the dashed lines are the upper and lower limit of agreement. **Figure S4.** Bland-Altman plots of the inter-observer agreement for oxyhemoglobin (OxyHb) and deoxyhemoglobin (DeoxyHb) measurements. The center line represents the mean difference, and the dashed lines are the upper and lower limit of agreement. a.u.: arbitrary units.
