# Supplementary figures and images for: Reliability assessment of hyperspectral imaging with the HyperView™ system for lower extremity superficial tissue oxygenation in young healthy volunteers
Source: J Clin Monit Comput. 2021 Apr 12;36(3):713–23. doi: 10.1007/s10877-021-00698-w (PMC9162963; doi:10.1007/s10877-021-00698-w)

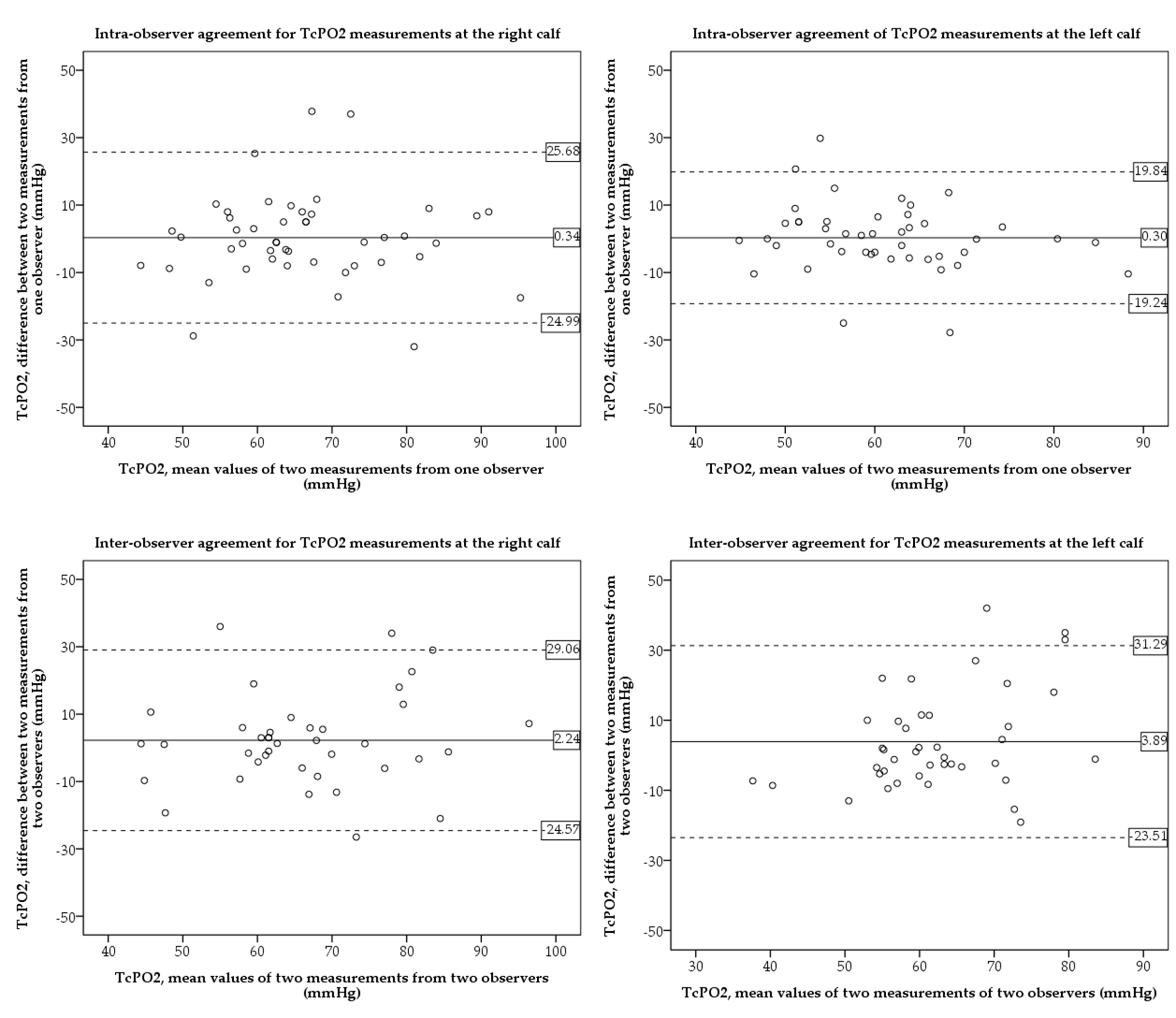

Supplement: Supplementary file 2 — Supplementary file2 (TIFF 725 kb) [file 10877_2021_698_MOESM2_ESM.tif]

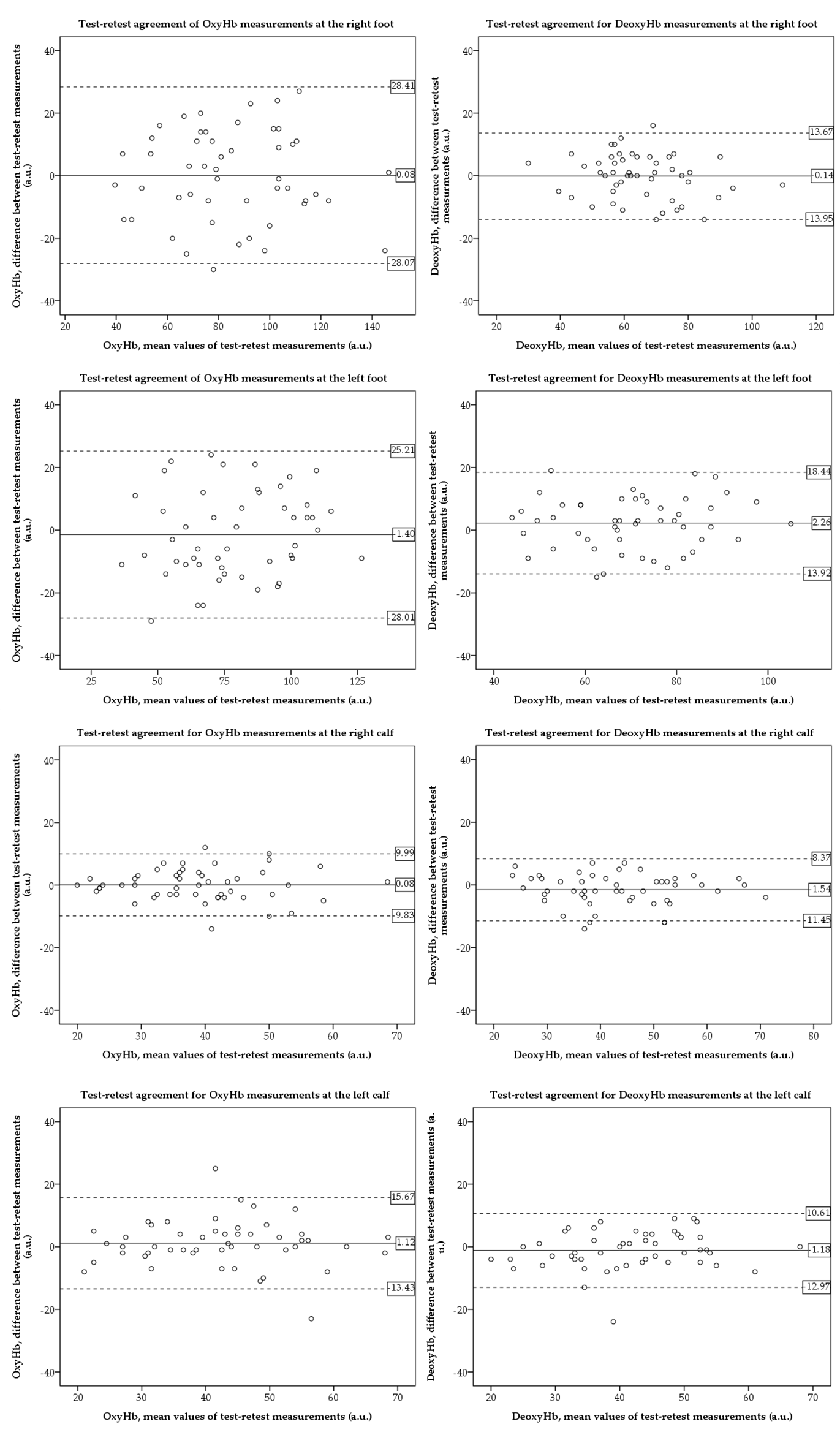

Supplement: Supplementary file 3 — Supplementary file3 (TIFF 499 kb) [file 10877_2021_698_MOESM3_ESM.tif]

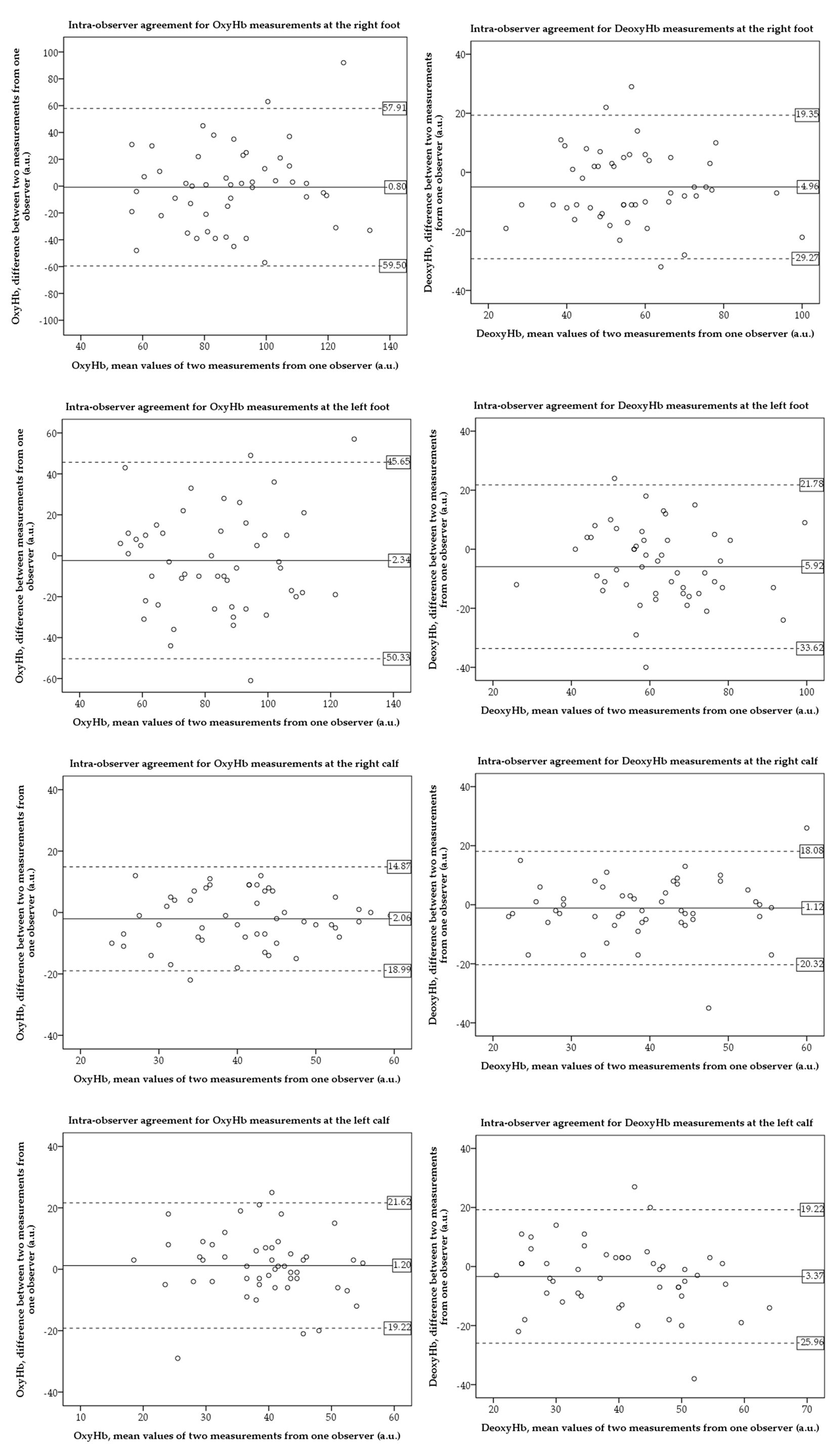

Supplement: Supplementary file 4 — Supplementary file4 (TIFF 783 kb) [file 10877_2021_698_MOESM4_ESM.tif]

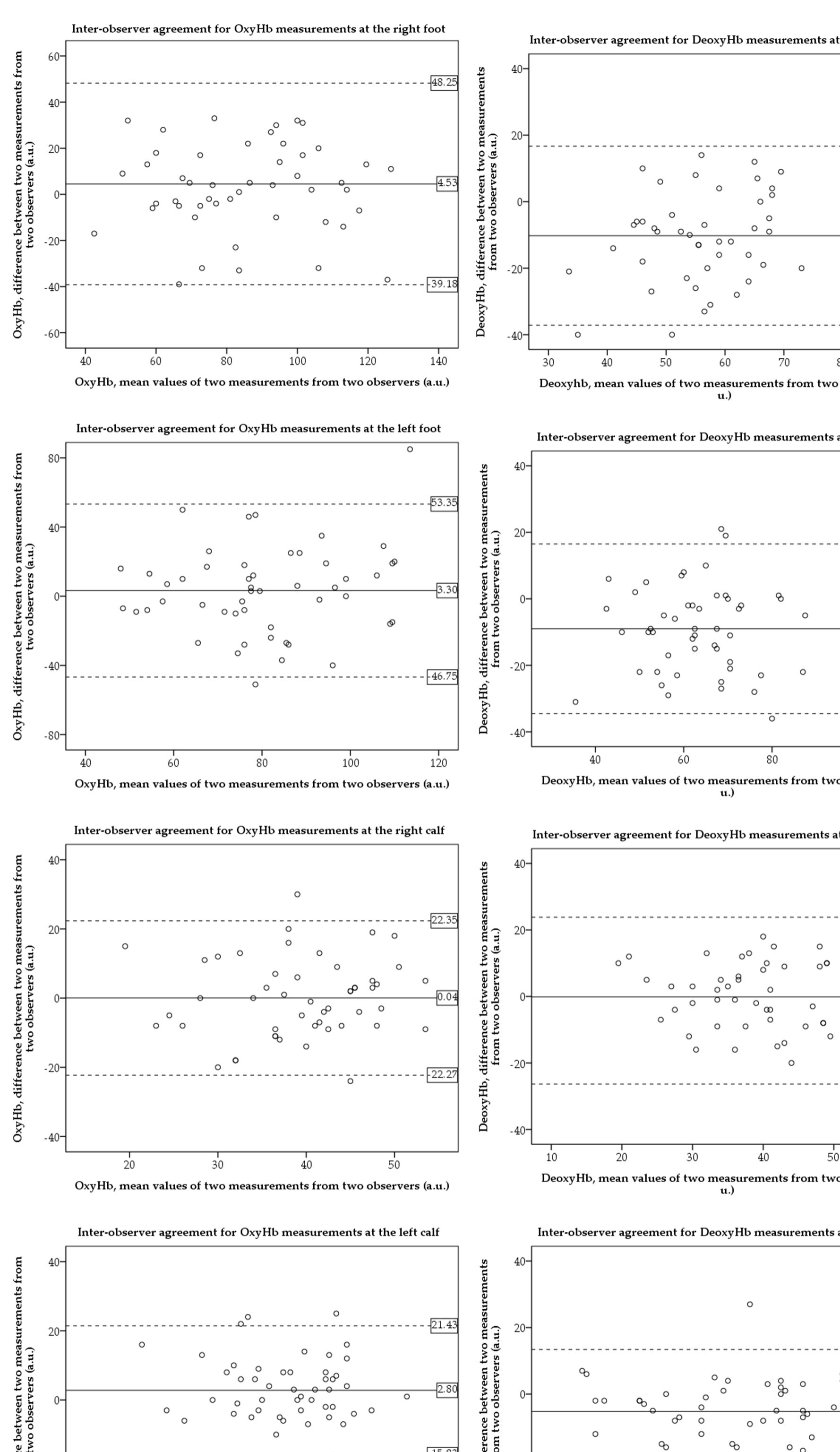

Supplement: Supplementary file 5 — Supplementary file5 (TIFF 870 kb) [file 10877_2021_698_MOESM5_ESM.tif]
